# Supplementary material for: New Solution for Segmental Assessment of Left Ventricular Wall Thickness, Using Anatomically Accurate and Highly Reproducible Automated Cardiac MRI Software
Source: J Imaging. 2025 Oct 11;11(10):357. doi: 10.3390/jimaging11100357 (PMC12565150; doi:10.3390/jimaging11100357)
Supplement: Supplementary file 1 [file jimaging-11-00357-s001.zip › Supplementary Material S1_OptiLayer short manual.pdf]

OptiLayer

An auxiliary application for accurate segmental measurement of compact myocardial wall thickness.

Name and purpose of the software:

OptiLayer: an auxiliary application for the Medis Suite software, designed for accurate segmental measurement of the compact myocardial layer (dense muscle tissue) thickness.

Medis Suite is an advanced cardiac MRI post-processing software specialized in cardiac assessment. It has been regularly used in our clinical practice for over 15 years. In Hungary, it is almost universally employed for this purpose, and internationally it ranks among the top 5 most widely used and recognized cardiac MRI evaluation tools.

The aim of OptiLayer is to measure myocardial thickness accurately in the appropriate segments, thereby revealing the location and extent of myocardial hypertrophy or thinning. Precise knowledge of these changes can have therapeutic implications in certain cardiomyopathies, such as hypertrophic or dilated cardiomyopathy. Additionally, the software holds research potential, for example, in risk assessment of left ventricular noncompaction.

Problem overview and algorithm:

According to internationally accepted guidelines, the left ventricle, which determines cardiac pump function, is divided into 17 segments: 6 basal segments (1-6), 6 mid-ventricular segments (7-12), 4 apical segments (13-16), and one apical cap segment (17). For wall thickness assessment, a modified 16-segment model is used, since the thickness of the 17th segment (apical cap) cannot be reliably measured. Abnormalities in wall thickness in different cardiac pathologies can be characterized by measuring the thickness of these segments, and their regional analysis is supported by appropriate grouping and averaging of segmental data.

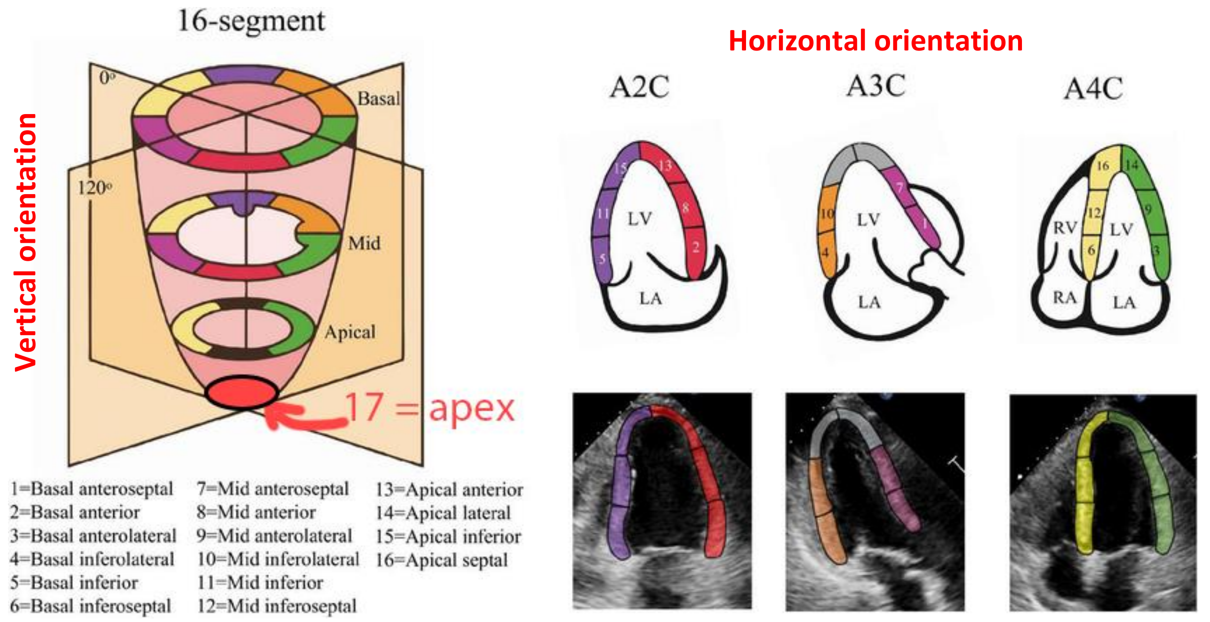

Original source of the edited image: Yinlong, Deng & Cai, Peiwei & Zhang, Li & Cao, Xiongcheng & Chen, Yequn & Jiang, Shiyan & Zhuang, Zhemin & Wang, Bin. (2022). Myocardial strain analysis of echocardiography based on deep learning. Frontiers in Cardiovascular Medicine. 9. 10.3389/fcvm.2022.1067760.

Currently, precise measurement of myocardial thickness is not feasible. Manual wall thickness measurement on cardiac MRI images is subjective and can show 2-3 mm differences within the same segment, leading to statistically unreliable results. This manual method shows considerable inter-operator variability and thus low reproducibility, which poses a significant problem for clinical decision-making. In contrast, the Medis software automatically performs 100 wall thickness measurements along the endocardial and epicardial contours on MRI slices. However, these measurements are divided into six parts using mathematical ratios rather than the actual anatomical segment boundaries. Because this sampling method is based on erroneous clinical assumptions and does not represent anatomical truth, Medis software itself is not suitable for determining segmental myocardial wall thickness.

OptiLayer was developed to provide precise wall thickness measurement within anatomically correct segmental boundaries, based on the 16-segment model. It calculates the average of automated measurements within these accurate segment borders. The software algorithm first identifies the apex (the 17th non-measured segment), which is manually marked as zero thickness on the Medis contour (endo- and epicardial contours are merged). OptiLayer detects this (<1.5 mm thickness values) and re-segments the remaining area from the apex towards the base, enabling anatomically valid segment positions. Lastly, the software averages the automatic measurements (12-13 per segment) within the new segment borders.

Target users:

This application is designed to process cardiac MRI data exported from Medis Suite, specifically supporting segmental evaluation of myocardial wall thickness. The software aims to provide clinicians and researchers in experimental settings with an efficient tool for assessing myocardial segments, thereby facilitating interpretation and clinical decision-making.

Key features:

In the first step, a semi-automatic, optimized contour must be created in Medis, excluding the apical segment (segment 17). This is achieved by fully extending the endocardial contour to meet the epicardial contour over approximately 1 cm in the apical segment. Next, the contour data should be saved as CSV files.

Myocardial segmentation into 16 segments for MR images is based on three imaging planes, which can be exported from Medis either as three separate files (each containing a single orientation), or as a single composite file (containing all three orientations).

Upon startup, the OptiLayer software opens a data input form for loading CSV files — either as one combined file or as three separate files, depending on how the export was performed. If the file names encode both the image orientation and sequence (such as “2ch”, “3ch”, “4ch”), the software recognizes these automatically; otherwise, the user can manually assign the correct orientation within the interface. Multiple patients can be added to OptiLayer at once and subsequently analyzed individually. Files may be loaded using the file browser button or by drag-and-drop. Erroneously added files can easily be removed.

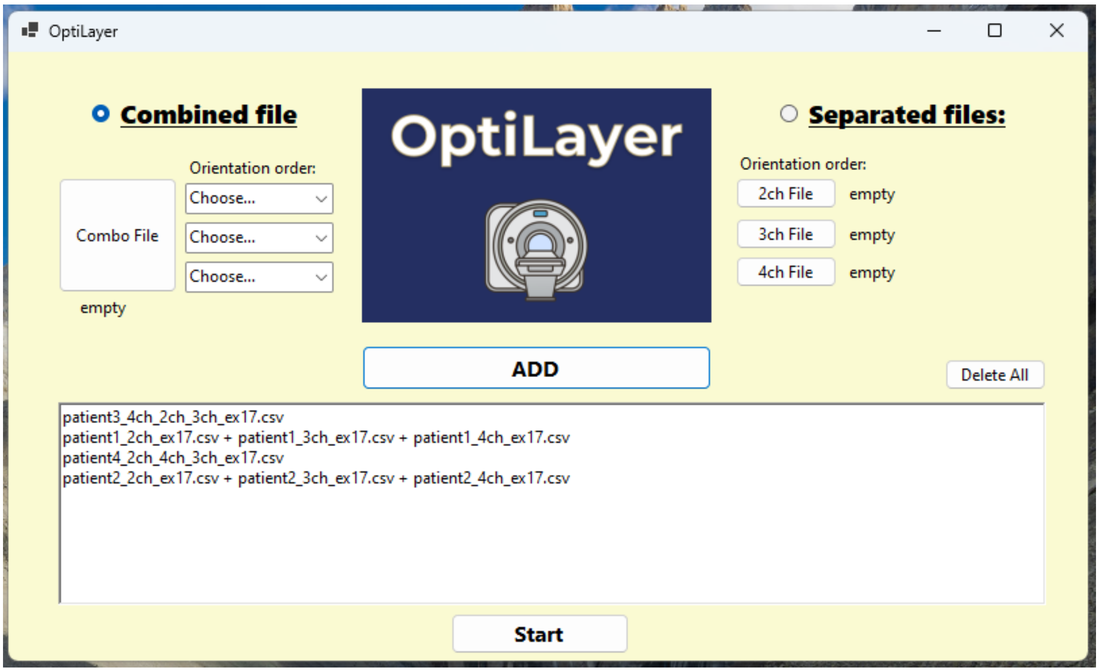

Initiating an evaluation launches a new form, allowing the user to select among the uploaded patients. The segmental myocardial wall thicknesses—reported in millimeters to two decimal places—are displayed on a "BullsEye" plot of the left ventricle. In this visualization, segments with normal thickness appear in green, those thinner than 5mm are shown in orange, and those thicker than 15mm appear in red. There are also options for horizontal and vertical filtering of segments.

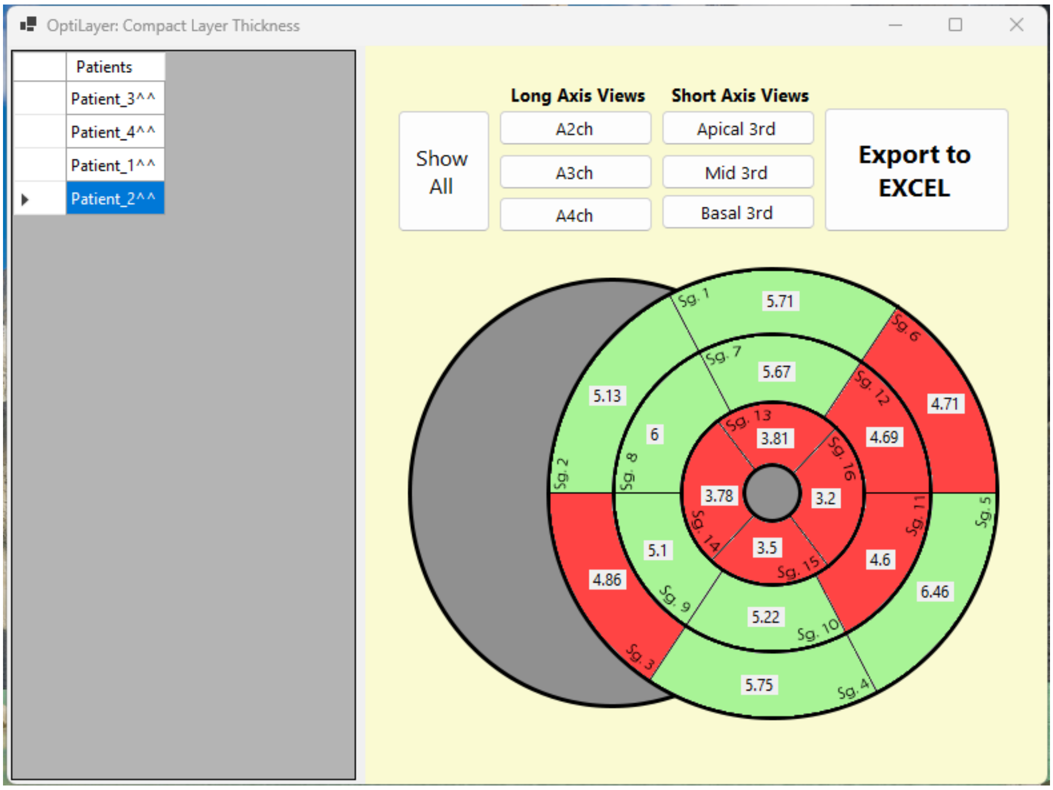

The "Export to Excel" button allows saving the wall thickness measurements generated by OptiLayer for a given patient as an .xlsx file. The exported Excel file contains the patient’s name, annotated segmental measurements, and average wall thickness values for the basal, mid-ventricular, and apical thirds. These data are useful both in the clinical setting and for research purposes.

Technologies and Platform Used:

The software is a Windows Forms application developed in C#, using Visual Studio 2022 (version 17.0) and runs on Windows 10+ with the .Net Framework 6.0. Development utilized the Medis Suite 4.0 and the integrated QMASS 8.1 automatic contouring and evaluation algorithm, but the software is compatible with any Medis Suite version from 3.0 onwards. Microsoft Office is recommended for reading the Excel exports.

Minimum system requirements:

- Operating system: Windows 10 (64-bit, version 1607 or later) or Windows 11
- Processor: 64-bit, minimum 1.6 GHz
- Memory (RAM): minimum 2 GB (4 GB recommended)
- Screen resolution: minimum 1024 x 768; 1920 x 1080 (Full HD) recommended
- Storage: at least 150 MB free disk space
- Required: .NET 6.0 Runtime (automatically downloaded at startup if missing)
